# Efficacy and safety of prophylactic intraoperative sac embolization in EVAR for abdominal aortic aneurysm: A meta-analysis

**DOI:** 10.3389/fsurg.2022.1027231

**Published:** 2023-01-06

**Authors:** Quan Chen, Yuan Zhang, Kangqing Lei, Liangyin Fu, Dengxiao Zhang, Wanli Sun, Chaohai Shi, Qibing Niu

**Affiliations:** ^1^ Department of Intervention and Vascular Surgery, Dongguan People's Hospital, Dongguan, Guangdong, China; ^2^ The First Clinical Medical College of Gansu University of Chinese Medicine(Gansu Provincial Hospital), Lanzhou, Gansu, China; ^3^ Department of Vascular Surgery, Gansu Provincial Hospital, Lanzhou, Gansu, China

**Keywords:** abdominal aortic aneurysm, endovascular aneurysm repair, embolization, type II endoleak, SAC

## Abstract

**Objective:**

We aimed to investigate the effectiveness and safety of prophylactic sac embolization during endovascular aneurysm repair (EVAR) in patients suffering from abdominal aortic aneurysms.

**Methods:**

We performed a systematic literature search of PubMed, Web of Science, EMbase, Cochrane Library, China National Knowledge Infrastructure (CNKI), VIP, Wanfang and China Biomedical Literature Database (CBM) to identify studies evaluating the outcomes of sac embolization vs. no embolization among patients who had received EVAR. The time limit of the search was from the establishing database to July 22, 2022. Outcome measures involved the type II endoleak rate, the other endoleak rate, the reintervention rate, mortality, and operation time. Fixed (no heterogeneity) or random effects models were constructed for each outcome. The outcomes are represented as the odds ratio (OR) with a 95% confidence interval (CI).

**Results:**

Among the 2,622 studies screened, 13 studies involving 747 participants were included in the review. The incidence of early-term type II endoleak (OR = 0.2, 95% CI (0.13,0.31), *P *< 0.00001), mid-term type II endoleak (OR* *=* *0.23, 95% CI (0.15,0.37), *P *< 0.00001), late-term type II endoleak (OR* *=* *0.27, 95% CI (0.16,0.46), *P *< 0.00001) and reintervention (OR* *=* *0.50, 95% CI (0.37,0.78), *P *= 0.002) within the sac embolization group were significantly lower than those in the non-embolization group. No significant differences were observed between the two groups were found for the other endoleak rates (OR* *=* *0.67, 95% CI (0.34,1.32), *P *= 0.25), mortality (OR* *=* *0.64, 95% CI (0.25,1.66), *P *= 0.36) and operation time operation (MD = 5.76, 95% CI (-8.30,19.83), *P *= 0.42).

**Conclusions:**

EVAR combined with sac embolization effectively reduces the incidence of type II endoleak and the reintervention rate without enhancing the operation time. Therefore, more high-quality studies are still needed for validation due to the limited amount and quality of included literature.

**Systematic Review Registration:**

https://www.crd.york.ac.uk/PROSPERO/, identifier: CRD42022365648.

## Introduction

An abdominal aortic aneurysm (AAA) is a typical aneurysmal arterial disease. Where anatomy permits, endovascular aneurysm repair (EVAR) has become an alternative to open surgery for AAA (1). Some international clinical studies have depictedt that the short-term mortality after EVAR is significantly lower than open surgery (2–5). However, the problem of endoleaks after EVAR is quite significant.

Type II endoleak is the most common type, detected in 6% to 59% of patients after EVAR (6, 7). Risk factors related to type II endoleak include age, smoking, the maximum diameter of the aneurysm, the inferior mesenteric artery (IMA), and the number of patent lumbar arteries (LA) (8). Multiple studies have shown a significant association between higher rates of type II endoleaks and preoperatively patent sac branches (IMA and LA) (9, 10). IMA is the inflow vessel of type II endoleak and LA is the outflow vessel. Patent vessels provide a complex array of inflow and outflow vessels, maintaining the blood flow and pressure within the sac (11). In addition, persistent type II endoleaks with increase in sac size could lead to new type I, type III endoleaks, or EVAR-related complications. They have a potential risk of rupture and a mortality rate of up to 80% during rupture (12, 13). Currently, the treatment of type II endoleaks mainly involves transarterial embolization of IMA or LA, direct translumbar embolization of the aneurysm sac, laparoscopic ligation of IMA and/or LA, or conversion to open surgery. However, the recurrence and subsequent re-intervention are possible with increased follow-up monitoring. It also enhancest the financial burden of the patients (14–16). Therefore, effective management of type II endoleaks is quite challenging.

Intraoperative sac embolization during EVAR can reduce the incidence of type II endoleaks. However, there is no consensus on the efficacy of intraoperative sac embolization. We screened the available data and performed a meta-analysis to evaluate the effectiveness and safety of prophylactic sac embolization during EVAR in AAA patients.

## Materials and methods

### Literature search

A systematic literature search involving PubMed, Web of Science, EMbase, Cochrane Library, China Knowledge Network (CNKI), Vipshop, Wanfang and the Chinese Biomedical Literature Database (CBM) was performed to retrieve randomized controlled trials (RCT) and retrospective cohort studies of prophylactic sac embolization in EVAR due to AAA. The search time limit was from establishing the database to July 22, 2022. The Chinese search terms included: “embolization”, “endoleak”, “aortic aneurysm, abdominal”, “abdominal aortic aneurysm”. The English search terms included: “Embolotherapy”, “Embolotherapies”, “Therapeutic Embolization”, “Embolizations, Therapeutic”, “Therapeutic Embolizations”, “Embolization”, “Endoleak”, “Endoleaks”, “Perigraft Leak”, “Leak, Perigraft”, “Leaks, Perigraft”, “Perigraft Leaks”, “Abdominal Aortic Aneurysms”, “Aneurysms, Abdominal Aortic”, “Aortic Aneurysms, Abdominal”, “Abdominal Aortic Aneurysm”, “Aneurysm, Abdominal Aortic”, “Aneurysm, Abdominal Aortic”.

### Inclusion and exclusion criteria

We included the studies depending on the following criteria: (1) Type of study: RCT or retrospective cohort study; (2) Subjects: AAA patients undergoing EVAR; (3) Intervention measures: the experimental group was treated with EVAR + intraoperative sac embolization, and the control group was treated with standard EVAR; (4) Outcome measures: incidence of type II endoleak, incidence of other endoleaks, reintervention rate, mortality, and operative time.

The exclusion criteria were:(1) Lack of control group; (2) Literature without primary outcome measures; (3) Duplicated studies; (4) Conference abstract; (5) Languages other than Chinese and English.

### Study selection and data extraction

Two evaluators independently screened the literature, extracted the data, and examined them. Any inconsistencies were carefully discussed, and a third party was consulted to resolve the issue. Based on inclusion and exclusion criteria, the initial screening was done by reading the “title and abstract” of the article to exclude any irrelevant studies. Then the full text was assessed to recruit the final literature. After confirming the inclusion, the following data were extracted: basic information about the literature (first author, publication date, type of study), age, gender, sample size, follow-up time, embolic criteria, embolic material, monitoring mode, outcome indicators, and the critical elements of risk of bias assessment.

### Statistical analysis

Statistical analysis was performed using RevMan 5.3 software. Continuous variables used the mean difference (MD) as the effect indicator, and dichotomous variables used the odds ratio (OR) with 95% confidence intervals (95% CI). They were calculated for each effect indicator and *P*-values, with *P *< 0.05 representing a statistically significant difference. Heterogeneity between studies was assessed using the *I*^2^ statistic with a cutoff value of 50%. If *I^2 ^*≤ 50%, there was no heterogeneity and a fixed effects model was used. On the other hand, if *I^2 ^*> 50%, a random effects model was used, and further heterogeneity analysis was performed through source line descriptive analysis. Two evaluators assessed three RCTs for risk of bias using the risk of bias assessment tool recommended in the Cochrane Handbook 5.1.0, and nine cohort studies were evaluated using the NOS quality rating scale.

## Results

### Literature search results

**Figure 1:**
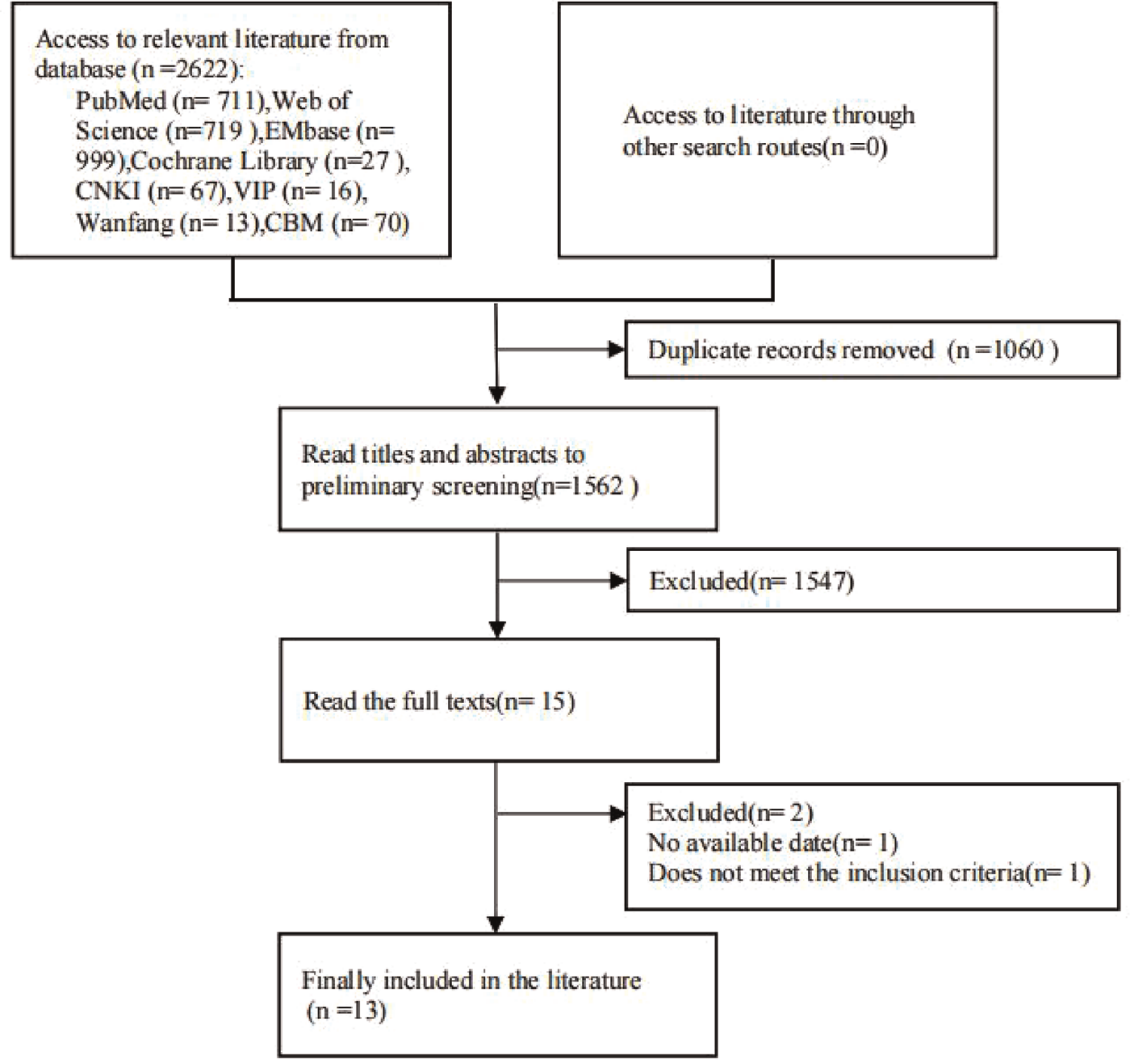
Flow diagram of meta-analysis.

A total of 2,622 articles were obtained through the database search. After removing duplicate studies and screening based on the inclusion and exclusion criteria, according to the PRISMA 2020 statement (17), we included 13 studies (18–30) with 747 patients, of which 330 underwent sac embolization during EVAR. The literature screening flow diagram and the results are depicted in Figure 1.

### Study and patient characteristics

The primary characteristics of the included studies are represented in Table 1, outcome measures of the included studies are demonstrated in Table 2, and the risk of bias assessment results of the RCT studies is shown in Table 3. The quality assessment of the cohort studies was performed with the Newcastle-Ottawa Scale (NOS), having with a total score of 9. The NOS scores of the included articles were ≥6, depicting high quality (Table 1).

**Table 1:** Baseline characteristics of the included studies.

Included studies	Type of study	Country	N (T/C)	Age (T/C)	Male (T/C)	Follow-up, months (T/C)	Surveillance	Embolic material	Inclusion criteria	NOS
Dosluoglu 2018 (18)	Review	America	16/31	70/72^a^	NR	44/44^a^	CT/DUS	Coils	High risk	8
Fabre 2021 (19)	RCT	France	47/47	72/73^a^	42/44	24/24^a^	CT/DUS	Coils	Regular	NR
Mascoli 2016 (20)	Review	Italy	26/44	73/72^a^	25/42	12/12^a^	DUS	Coils	High risk	7
Miura 2020 (21)	Review	Japan	83/93	76/76^a^	68/85	12/12^a^	CT	NBCA	Regular	6
Natrella 2017 (22)	Review	Italy	36/36	79/77^b^	28/33	12/12^a^	CT/DUS	Fibrin glue + Coils	Regular	8
Ohba 2020 (23)	Review	Japan	26/7	78/88^a^	18/6	14/6.5^b^	CT	NBCA	rAAA	6
Piazza 2013 (24)	Review	Italy	79/83	71/71^a^	72/73	13.2/37.2^a^	CT	Fibrin glue + Coils	Regular	6
Piazza 2016 (25)	RCT	Italy	50/55	75/76^a^	48/52	12/12^a^	CT	Fibrin glue + Coils	High risk	NR
Pilon 2010 (26)	Review	Italy	18/20	72/72^a^	NR	18.5/20^a^	CT	Fibrin glue + Coils	Regular	6
Ronsivalle 2010 (27)	Review	Italy	180/224	73/72^a^	161/210	26/72^b^	CT/DUS	Fibrin glue + Coils	Regular	8
Chen HY 2018 (28)	Review	China	33/36	74/70^b^	24/28	12/12^a^	CT	Coils	Regular	6
Li XT 2019 (29)	Review	China	36/76	NR	NR	25.9/29.4^a^	CT/DSA	Fibrin glue + Coils	High risk	7
Zhou YR 2020 (30)	RCT	China	45/45	64/66^a^	22/24	12/12^a^	CT	Coils	Regular	NR

*T*, test group; C, control group; N, sample size; NR, not reported.

^a^ average.

^b^ median; rAAA, ruptured abdominal aortic aneurysm; NOS, newcastle-ottawa scale.

**Table 2:** Outcome measures of the included studies.

	Type II endoleak (T/C)			T/C				
Included studies	Early	Middle	Late	Other endoleak	Mortality	Re-intervention	Operation time, minutes	Radiation time, minutes
Dosluoglu 2018 (18)	1/15	NR	NR	0/5	4/7	2/12	135 ± 67/123 ± 59	NR
Fabre 2021 (19)	2/15	5/15	3/8	NR	1/1	2/8	NR	28. ± 7.6/21.4 ± 9.9
Mascoli 2016 (20)	7/30	5/32	NR	NR	1/0	NR	NR	NR
Miura 2020 (21)	2/21	NR	NR	NR	0/5	0/3	NR	NR
Natrella 2017 (22)	NR	2/9	NR	1/1	0/0	NR	NR	NR
Ohba 2020 (23)	NR	4/0	NR	NR	1/3	2/0	134 ± 50/98 ± 15	NR
Piazza 2013 (24)	8/20	NR	NR	2/3	NR	5/11	179 ± 49/185 ± 52	25.0 ± 7.3/26 ± 5.2
Piazza 2016 (25)	8/17	7/16	7/9	NR	5/2	3/8	157 ± 40/149 ± 51	23.5 ± 7.0/22.1 ± 6.5
Pilon 2010 (26)	NR	NR	1/6	NR	0/2	1/4	187 ± 37/207 ± 54	32.3 ± 13.1/30.0 ± 8.2
Ronsivalle 2010 (27)	NR	NR	4/34	6/13	20/21	14/25	NR	NR
Chen HY 2018 (28)	NR	2/9	NR	4/2	NR	NR	NR	NR
Li XT 2019 (29)	NR	4/25	4/23	0/1	NR	1/1	148 ± 54/142 ± 54	28.8 ± 5.0/25.8/6.8
Zhou YR 2020 (30)	1/5	2/10	NR	NR	0/9	NR	NR	NR

**Table 3:** Inclusion of RCT study bias risk assessment.

Included studies	Random method	Assign hidden	Blindness	Data integrity	Selective reporting of research findings	Other sources of offsets
Fabre 2021	Computer Random	NR	No	Yes	No	NR
Piazza 2016	Random Envelope Method	NR	NR	Yes	No	NR
Zhou YR 2020	Random Number Table Method	NR	NR	Yes	No	NR

### Type II endoleak incidence

**Figure 2:**
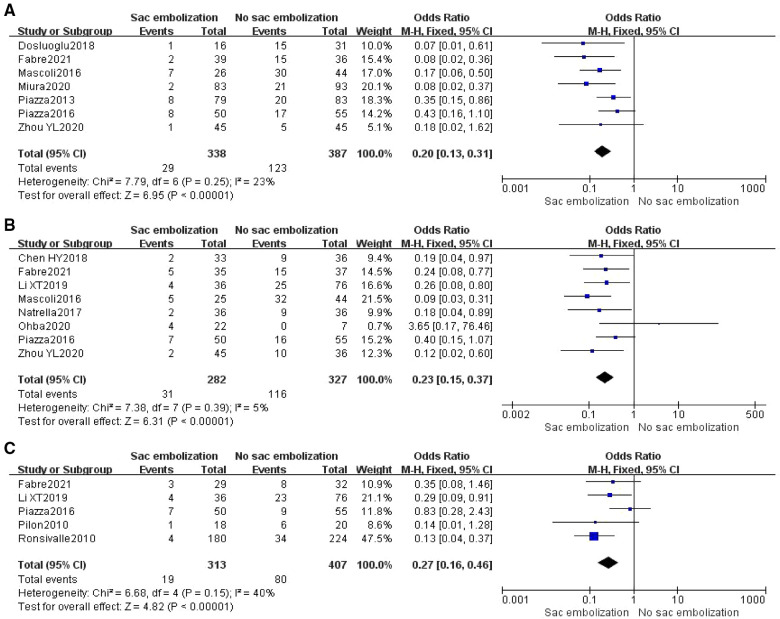
Incidence of early-term (**A**), mid-term (**B**), and late-term (**C**) type II endoleak in the embolization group compare with the non- embolization group.

Based on the follow-up time, the incidence of type II endoleaks was defined at 0–6 months, 7–18 months, and >18 months postoperatively as early, middle and late endoleaks. Seven studies reported the occurrence of early-term type II endoleak, and the meta-analysis indicated that the incidence of early-term type II endoleak was significantly lower within the embolization group than in the non-embolization group [OR = 0.2, 95% CI (0.13, 0.31), *P *< 0.00001] (Figure 2A). Eight studies reported the occurrence of mid-term type II endoleak, and the results revealed that the incidence of mid-term type II endoleak was significantly lower within the embolization group than in the non-embolization group [OR = 0.23, 95% CI (0.15, 0.37), *P *< 0.00001] (Figure 2B). Five articles reported the occurrence of late endoleaks, and meta-analysis showed that the incidence of late type II endoleak was significantly lower in the embolization group than in the non- embolization group [OR = 0.27, 95% CI (0.16, 0.46), *P* < 0.00001] (Figure 2C).

**Figure 3:**
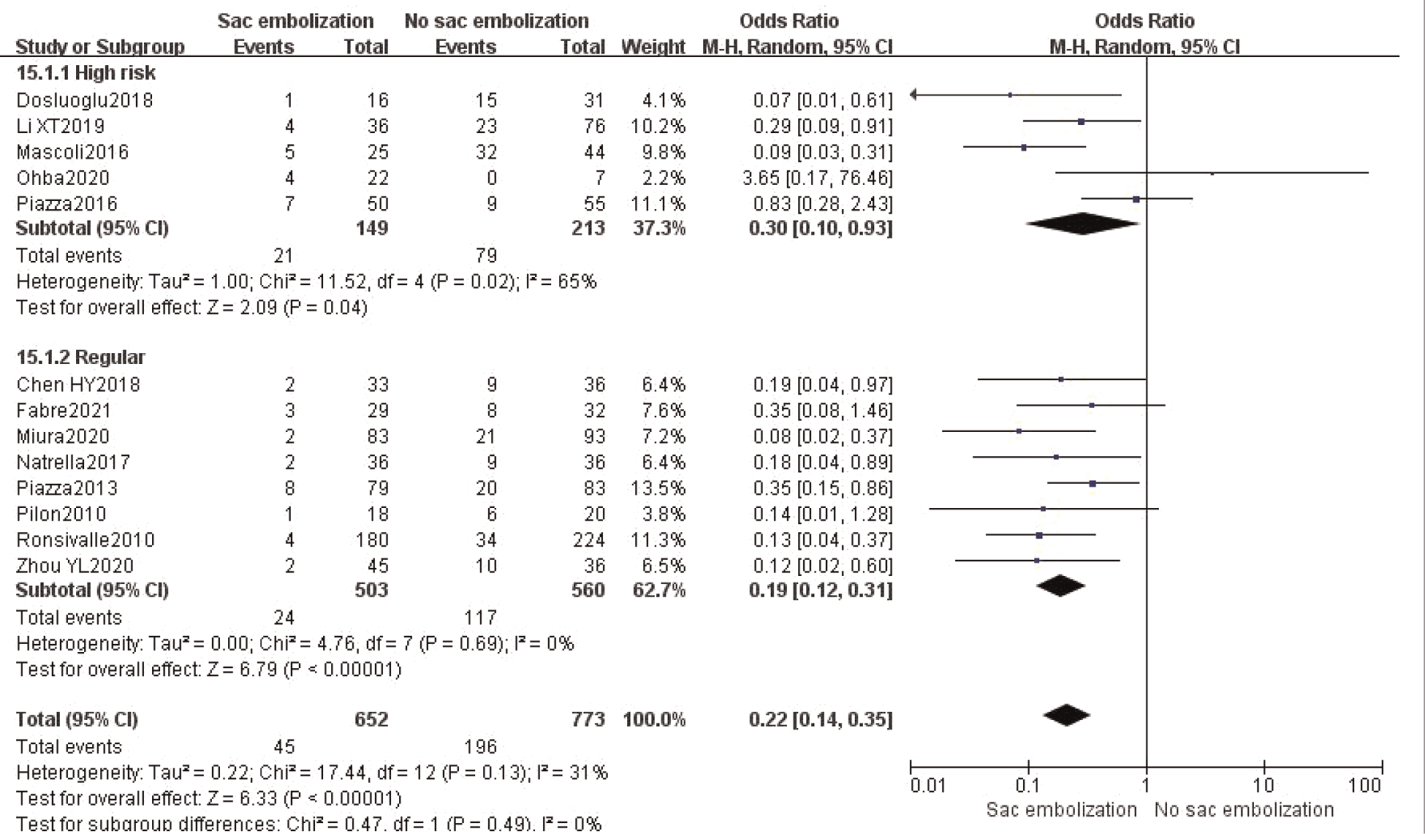
Incidence of type II endoleak at the last follow-up in the high risk group compare with the regular group.

Based on the inclusion criteria, subgroup analysis revealed that sac embolization could significantly decrease the rate of type II endoleak during the final follow-up among the regular and high-risk groups (*P *< 0.05) (Figure 3).

**Figure 4:**
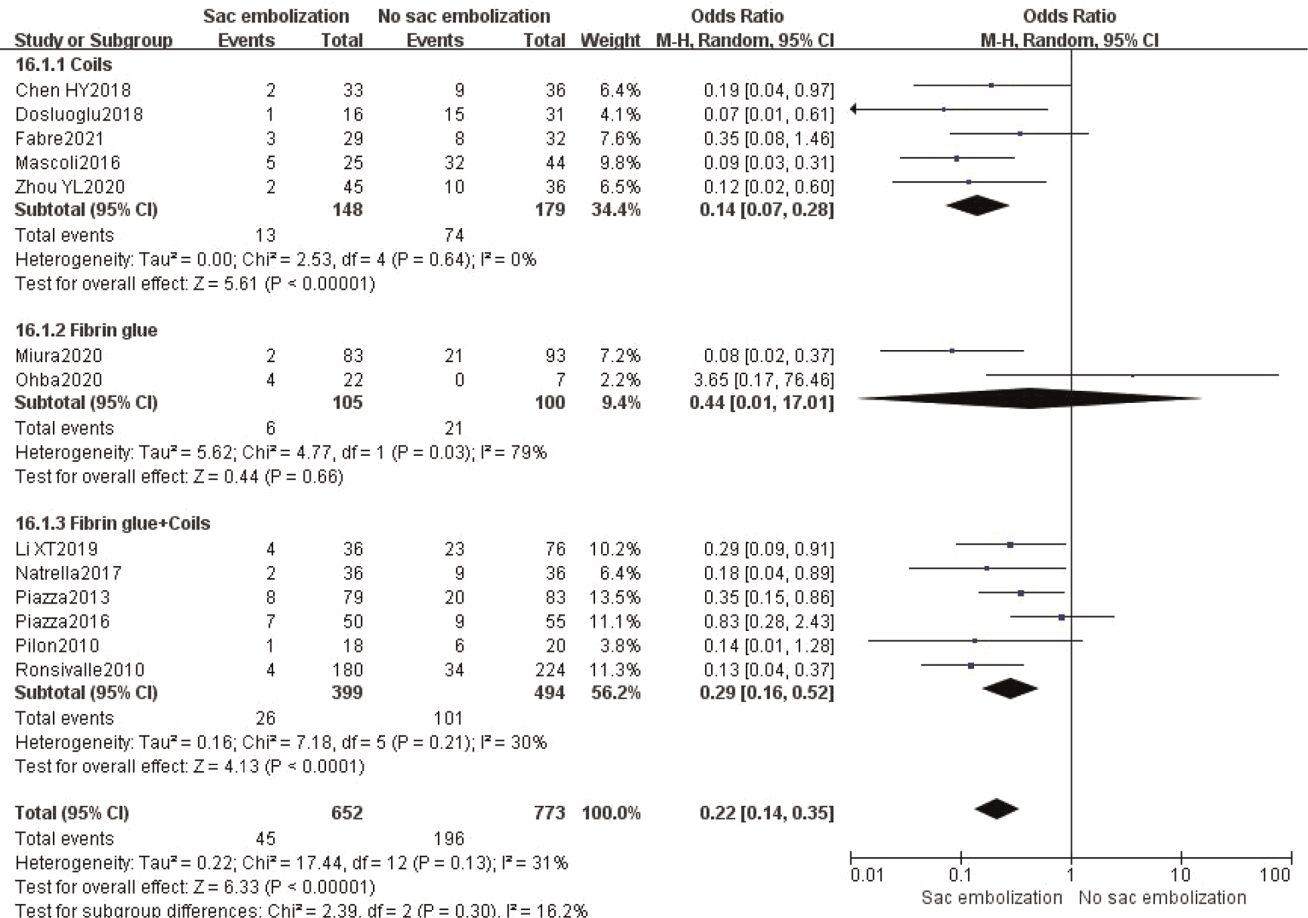
Incidence of type II endoleak at the last follow-up in different embolic material group.

According to the embolic material, subgroup analysis depicted that using coils and fibrin glue + coils could significantly reduce the type II endoleak rate during the final follow-up (*P *< 0.01). However there was no statistically significant difference in using fibrin glue alone groups [OR = 0.44, 95% CI (0.01, 17.01), *P *= 0.66] (Figure 4).

### Incidence of other endoleaks

**Figure 5:**
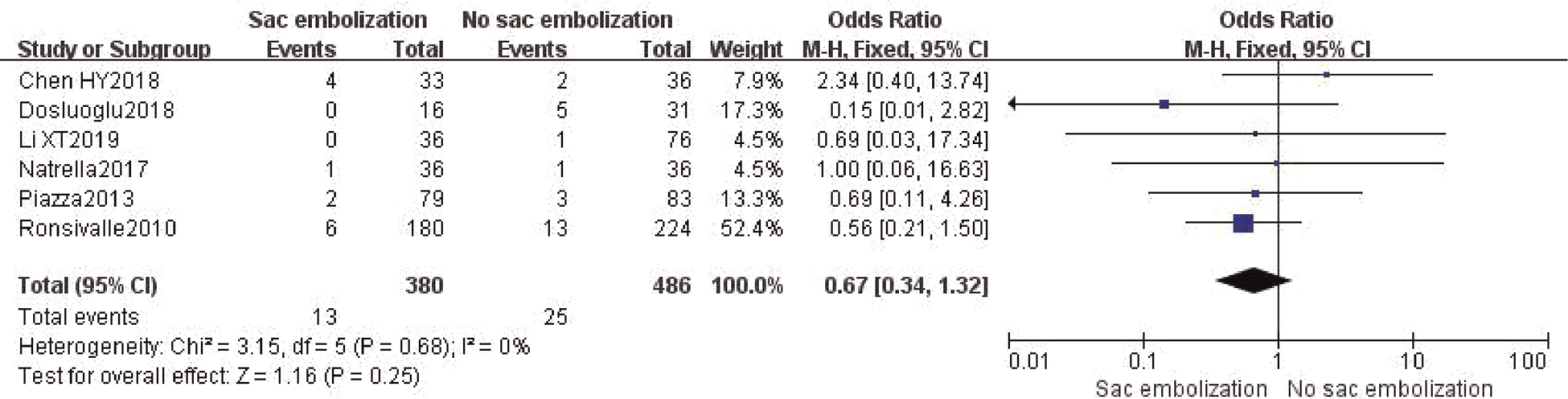
Incidence of other endoleaks in the embolization group compare with the non- embolization group.

Other endoleaks included type I, type III or type IV. Six studies described the occurrence of other endoleaks. The results depicted no statistically significant difference during incidence of other endoleaks between the embolization and the non- embolization groups [OR = 0.67, 95% CI (0.34, 1.32), *P* = 0.25] (Figure 5).

### Mortality

**Figure 6:**
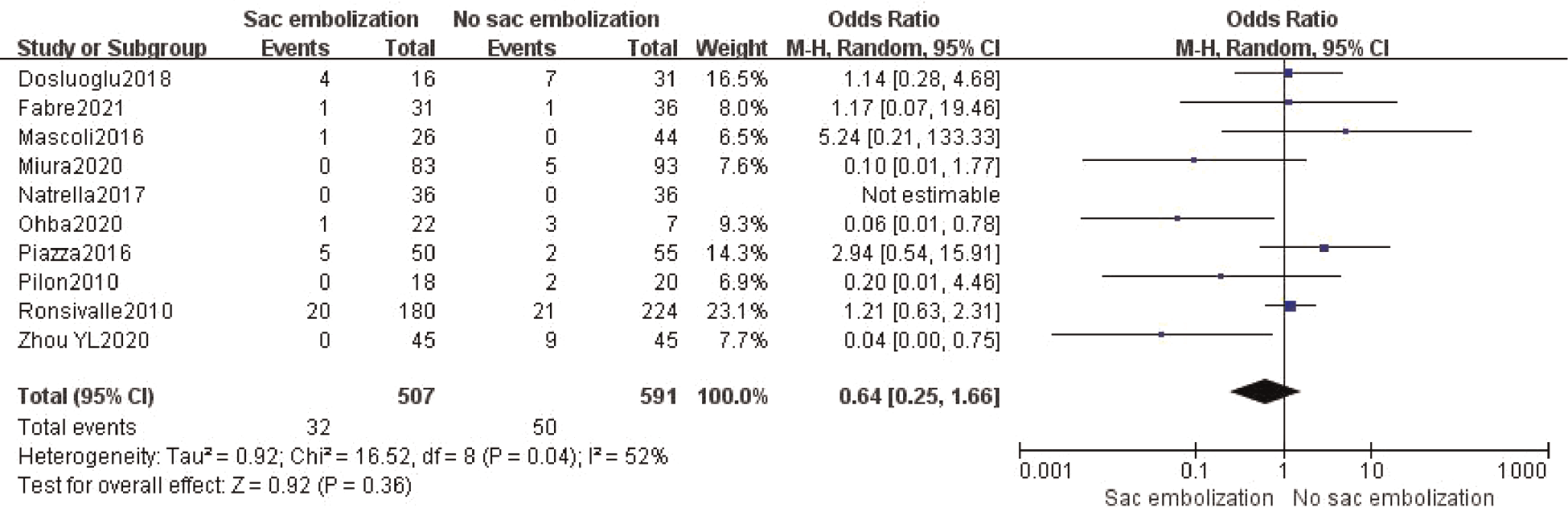
Mortality in the embolization group compare with the non- embolization group.

Ten studies reported the occurrence of mortality. The meta-analysis described no statistically significant difference in mortality among the embolization and non-embolization groups in the random effects model [OR = 0.64, 95% CI (0.25, 1.66), *P *= 0.36] (Figure 6).

### Re-intervention

**Figure 7:**
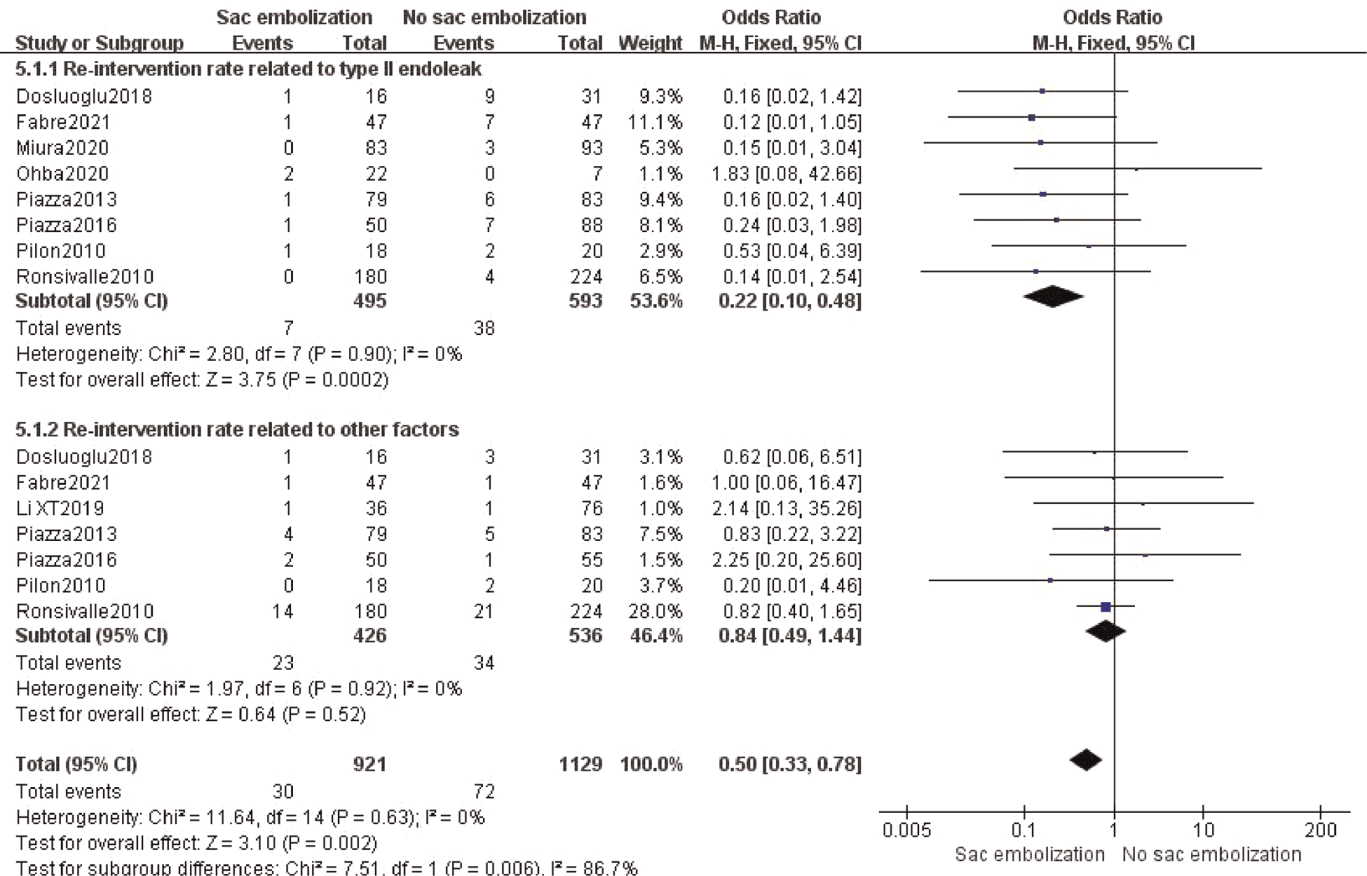
The rate of re-intervention in the embolization group compare with the non- embolization group.

Nine studies reported re-intervention, and meta-analysis showed that the re-intervention rate was lower in the embolization group compared to the non-embolization group, indicating a statistically significant difference [OR = 0.50, 95% CI (0.37, 0.78), *P *= 0.002]. Subgroup analysis revealed that the re-intervention rate associated with type II endoleak was significantly lower in the embolization group than in the non-embolization group [OR = 0.22, 95% CI (0.10, 0.48), *P *< 0.01]. However, there was no statistically significant difference in the re-intervention rate associated with other factors in the two groups [OR = 0.84, 95% CI (0.49, 1.44), *P *= 0.52] (Figure 7).

### Operation time

**Figure 8:**
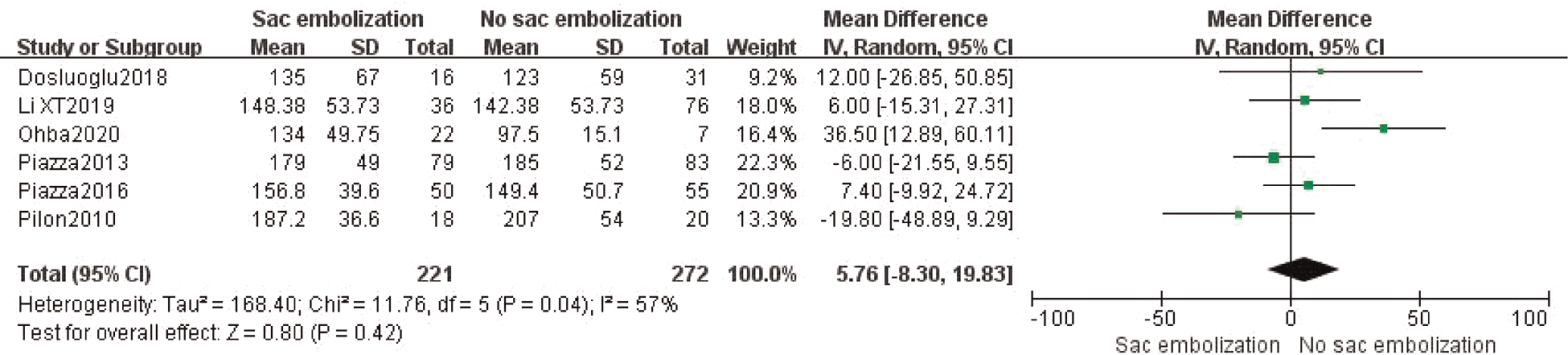
The time of operation in the embolization group compare with the non- embolization group.

Six studies reported the operation time, and the random effect model analysis showed no statistically significant difference in operation time between the embolization and the non-embolization groups [MD = 5.76, 95% CI (−8.30, 19.83), *P *= 0.42] (Figure 8).

### Publication bias

**Figure 9:**
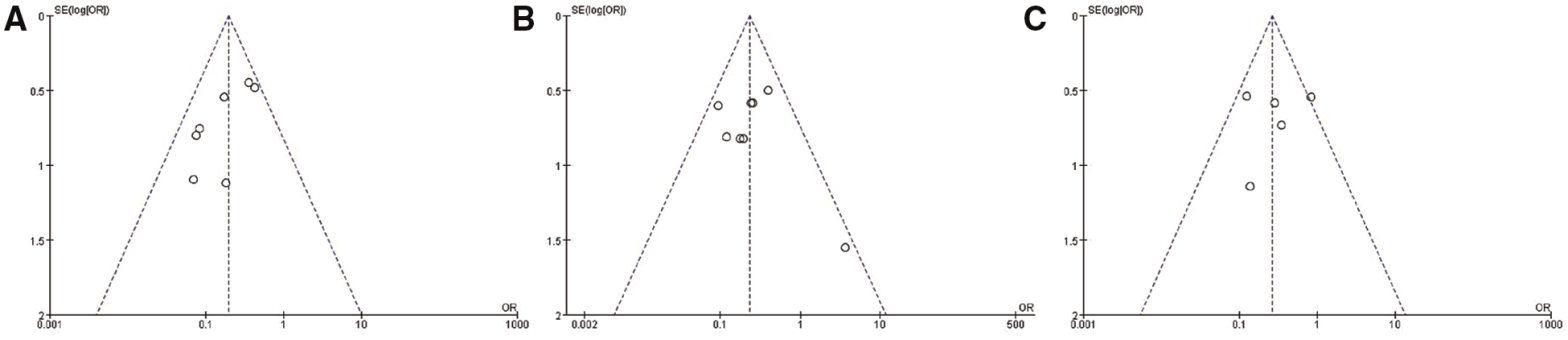
Publication bias (according to incidence of early-term (**A**), mid-term (**B**), and late-term (**C**) type II endoleak).

Funnel plots were created using RevMan 5.3 software depending on the incidence of early, middle, and late-term type II endoleaks. It showed substantial symmetry on both sides, establishing a low publication bias for inclusion (Figure 9).

### Discussion

EVAR has the advantages of less trauma, lower anesthetic requirements, and faster postoperative recovery compared to traditional open surgery. However, managing the postoperative EVAR sac is extremely important, and the reduction in aneurysm diameter during follow-up is an important marker for successful EVAR (31). Type II endoleak is the most common complication after EVAR. Some type II endoleaks may close or increase the diameter of the aneurysm sac and re-intervention (19). Sac embolization in EVAR is performed by developing new access on the opposite side of the delivery route of the main stent, thereby leaving the 4F contrast catheter within the AAA cavity. After successfully undergoing the standard EVAR, the coils or liquid embolization agent was delivered from the pre-positioned 4F catheter within the sac.

This study showed that EVAR combined with sac embolization could significantly reduce the incidence of type II endoleak in the early, middle, and late-term postoperative periods (*P *< 0.01). There was no significant effect on the incidence of other endoleaks (type I, III, and IV endoleaks) (*P *= 0.25). EVAR combined with sac embolization could also significantly decrease the re-intervention rate (*P *= 0.0007). Regarding safety, there was no significant difference in mortality and operation time between the sac embolization and the non- embolization groups.

In previous studies, different centers have reported different efficacy of sac embolization due to various follow-up times. The early-term effectiveness of EVAR combined with sac embolization has been widely recognized (18, 19, 25, 30) and confirmed by our studies. Pilon (26)and Ronsivalle (27) reported significant long-term efficacy. In an RCT study in 2021, Fabre et al. (19) reported that sac embolization prevented type II endoleaks at 1 month, 6 months, and 12 months. However, no statistically significant difference was observed in the incidence of type II endoleaks between the two groups at 24 months (*P *= 0.19). Piazza et al. (25) also described no significant difference in the rate of type II endoleaks among the two groups at 24 months after surgery (*P *= 0.57). Our study confirmed the efficacy of EVAR combined with sac embolization, significantly reducing the incidence of type II endoleaks during the middle and late term.

Thirty-eight cases of other endoleaks were reported in six studies, including 13 from the embolization group (11 cases of type I endoleaks and two from of type III endoleaks), and 25 from the non-embolization group (21 cases of type I endoleaks, one of type III endoleak and three of type IV endoleaks), with no statistically significant difference between the two groups (*P *= 0.25). A separate analysis for the type I endoleak incidence in the two groups also showed no statistically significant difference [OR = 0.67, 95% CI (0.33, 1.38), *P* = 0.28].

The impact of type II endoleaks on survival is remains unclear (18). Seike et al. (32) retrospectively analyzed the clinical data of 17,099 patients and show a correlation between type II endoleaks patients and late adverse events, including re-intervention, death, rupture, and aneurysm sac enlargement. Batt et al. (33) reported that survival with or without type II endoleak (T2E) had no statistical difference (*P* = 0.49). Sidloff et al. (34) found that a late type II endoleak was associated with survival (*P* = 0.008) but early type II endoleak was not (*P *= 0.06). Our study found that intraoperative embolization significantly reduced the incidence of postoperative type II endoleaks. Moreover, there was no significant difference in postoperative mortality between the two groups (*P *= 0.36), demanding confirmation through various RCTs.

In a study of 3,595 patients, van Marrewijk et al. (35) observed that 55% of patients with type II endoleaks required re-intervention, significantly higher than the 15% re-intervention rate among patients without type II endoleak. In our analysis, embolization effectively decreased the re-intervention rate (*P *= 0.002), and subgroup analysis revealed that embolization significantly reduced the re-intervention rate associated with type II endoleaks (*P *< 0.01). For re-intervention due to other reasons, there was no significant difference between the embolization and the non-embolization groups (*P *= 0.52). An issue with the embolization procedure is the potential for prolonging of operative time and radiation exposure, in our analysis, sac embolization did not extend the operative time (*P *= 0.42).

The study was conducted by following the methods and requirements of meta-analysis. However, there are certain limitations: (1) the sample size of some of the RCTs or retrospective cohort studies included in this study was small and required expansion; (2) lack of standardized in the treatment of study methods, such as lack of blinding and propensity matching, which could be biased; (3) different centers included their studies based on different prevention criteria, embolic materials and re-intervention criteria, which could affect the analysis analysis.

## Conclusion

In conclusion, compared with standard EVAR, EVAR combined with intraoperative sac embolization significantly decreases the incidence of postoperative type II endoleak and re-intervention rate but does not enhance the operation time. This study is limited by the quantity and quality of the included literature. Therefore, more high-quality studies are needed to validate the results.

## Data Availability

The original contributions presented in the study are included in the article/Supplementary Material, further inquiries can be directed to the corresponding author/s.
